# Supplementary figures and images for: ETS transcription factor pointed controls germline survival in Drosophila
Source: PLoS Genet. 2025 Aug 25;21(8):e1011809. doi: 10.1371/journal.pgen.1011809 (PMC12407541; doi:10.1371/journal.pgen.1011809)

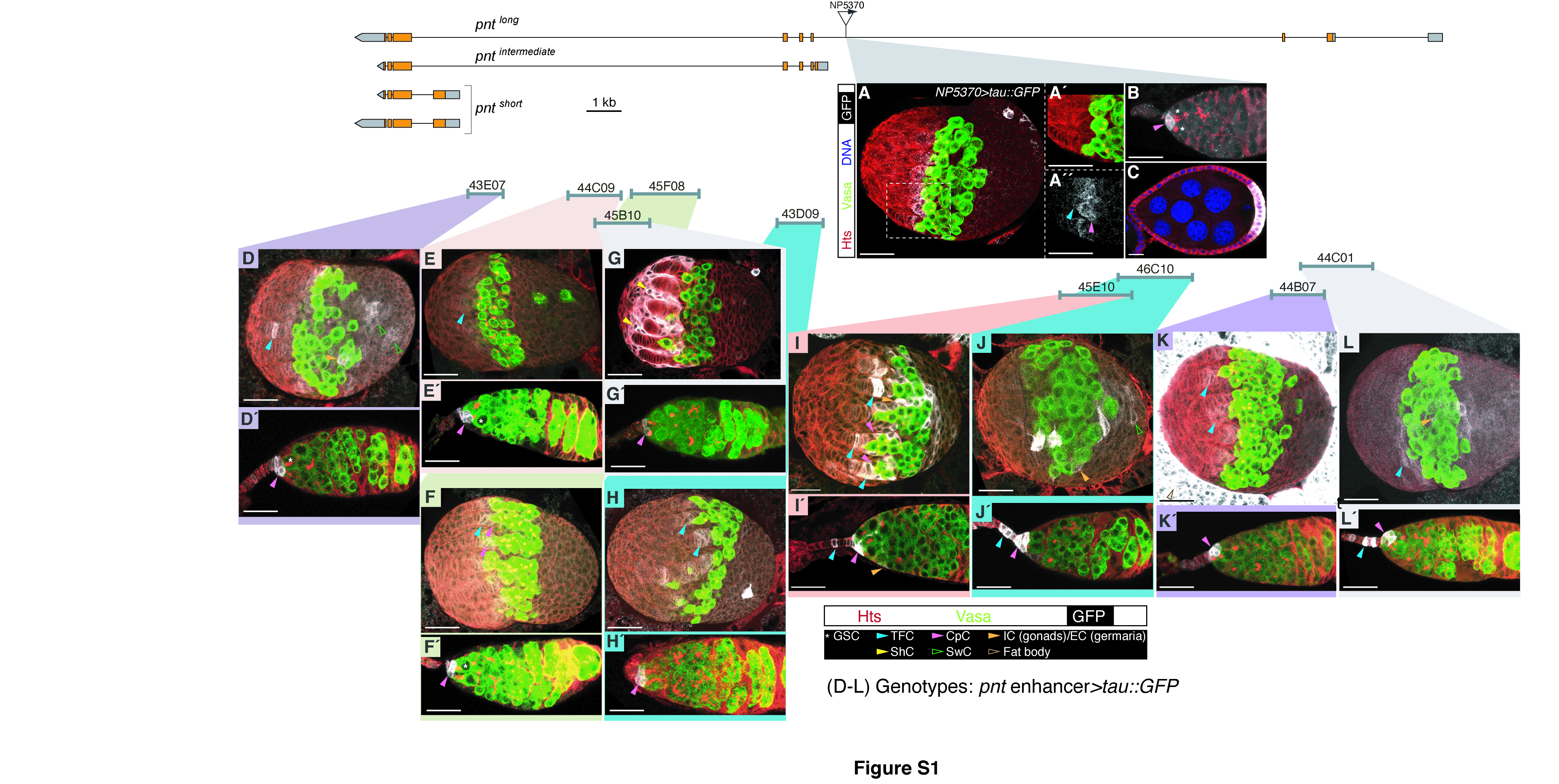

Supplement: S1 Fig — In all cases, the regulatory elements drive expression of the Gal4 transcriptional activator. As a reporter, we used the UASp-tau::GFP construct. (A-C) LL3 gonad, germarium and stage 6 egg chamber of NP5370 > tau::GFP female larva and adult. They have been stained with anti-Hts (red; to visualise cell outlines and germline spectrosomes and fusomes), anti-Vasa (green; to label the germline), anti-GFP (white; to show Tau::GFP localization) and with a DNA dye (blue; to mark chromatin). The molecular mapping of the NP5370 insertion is represented in the genomic map above the set of panels. (D-L) LL3 gonads and germaria of nine different pnt enhancer>tau::GFP female larvae and adults. They have been stained with anti-Hts (red), anti-Vasa (green) and anti-GFP (white). GSC: germline stem cell; TFC: terminal filament cell; CpC: cap cell; IC: intermingled somatic cell; EC: escort cell; ShC: sheath cell; SwC: Swarm cell. Scale bars: 20 μm. Related to Fig 1. (TIF) [file pgen.1011809.s001.tif]

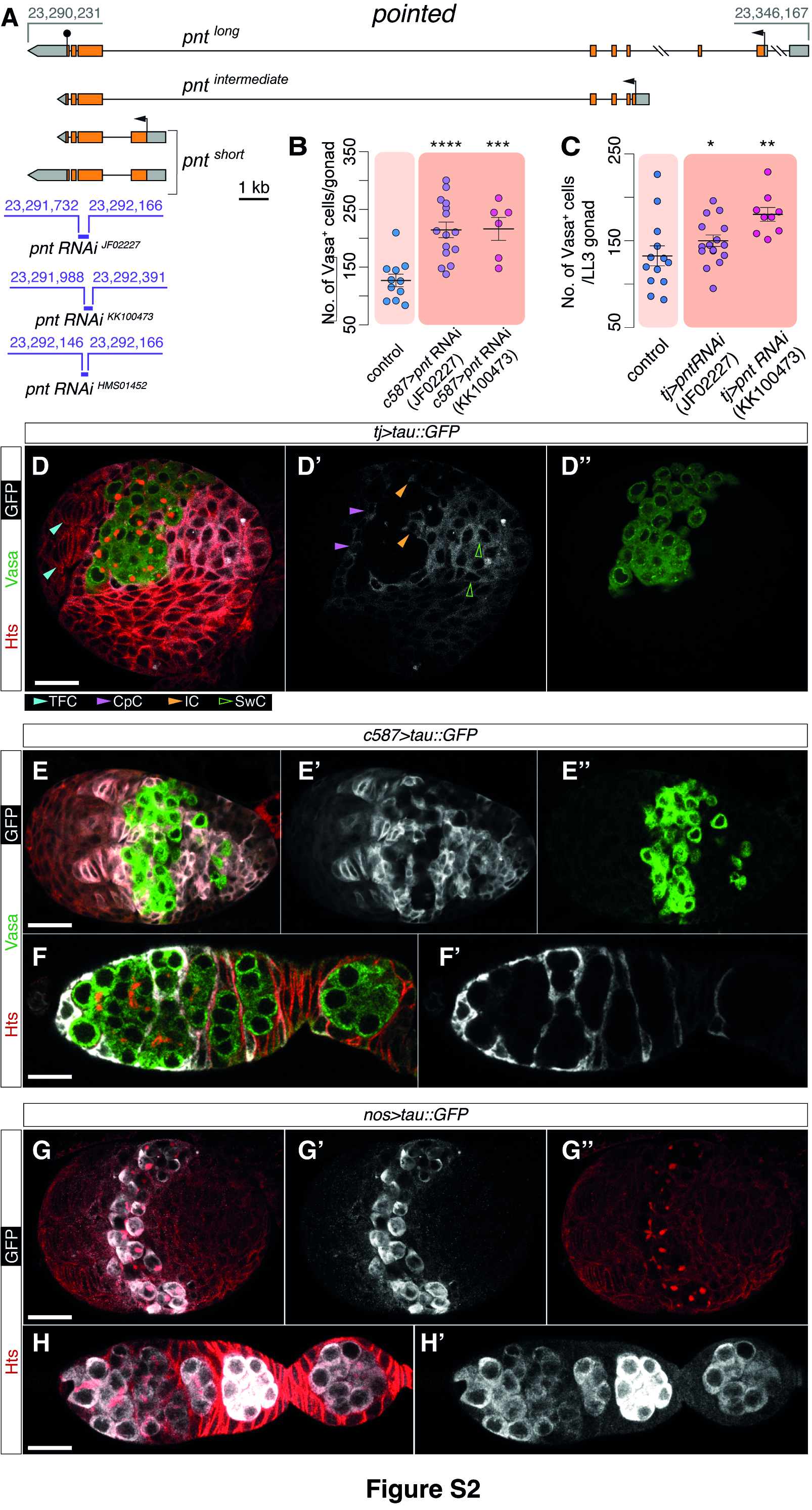

Supplement: S2 Fig — (A) Representation of the exon-intron organization of the pnt locus. The three RNAi lines used in this work target similar fragments of the coding region common to all of the transcripts. Numbers of genomic coordinates according to FlyBase (release FB2024_02). (B) Quantification of the number of Vasa+ cells in control, c587 > pnt RNAiJF02227 and c587 > pnt RNAiKK100473 LL3 gonads. (C) Quantification of the number of Vasa+ cells in control, tj > pnt RNAiJF02227 and tj > pnt RNAiKK100473 LL3 gonads. The arithmetic mean and the SEM are shown for each of the genotypes. (D) Pattern of expression of the tj-Gal4 line in control gonads visualised by the distribution of the Tau::GFP reporter (white). (E, F) Pattern of expression of the c587-Gal4 line in control LL3 gonads and adult germaria as shown by the Tau::GFP reporter. The localization of the Hts (red) and Vasa (green) proteins is used to outline cell shapes and to label the germline, respectively. (G, H) Pattern of expression of the nos-Gal4 line in control LL3 gonads and adult germaria as shown by the Tau::GFP reporter. The localization of Hts (red) is used to outline cell shapes. * = p < 0.05; ** = p < 0.005. Scale bar: 20 μm. Related to Fig 2. (TIF) [file pgen.1011809.s002.tif]

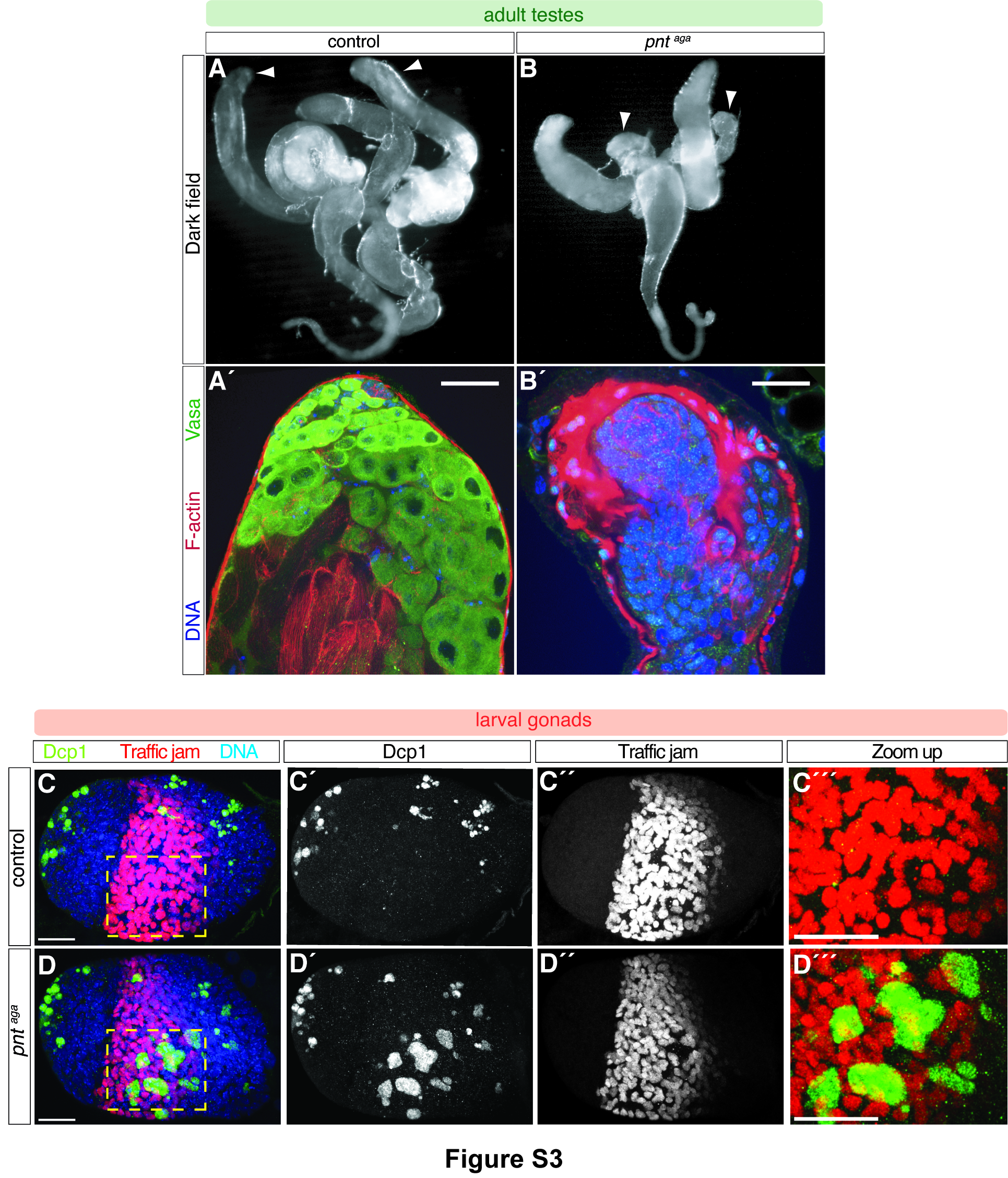

Supplement: S3 Fig — (A, B) dark field images of control (A) and pntaga (B) testes. (A’, B’) Control (A’) and pntaga (B’) testes stained to visualize F-actin (red) and Vasa (green; to label the germline). pntaga testes are devoid of germline cells. Scale bars: 20 μm. Related to Fig 3. (C) Control and (D) pntaga LL3 gonads stained with anti-Dcp1 (green; to visualise apoptotic cells), anti-Traffic Jam, anti-Vasa (red; to visualize IC) and with a DNA dye (blue). (TIF) [file pgen.1011809.s003.tif]

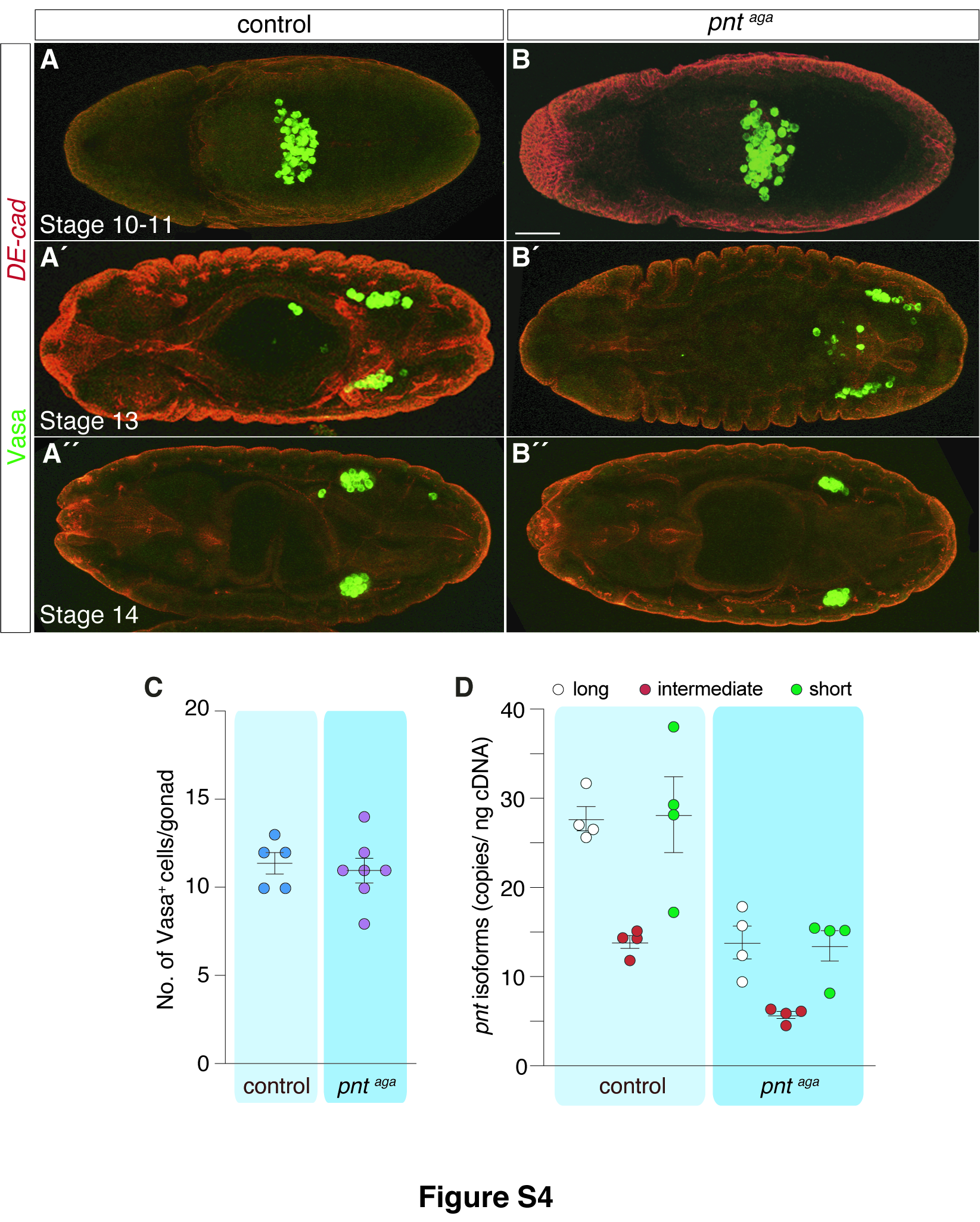

Supplement: S4 Fig — (A, B) Control (A) and pntaga (B) embryos at different stages of embryogenesis stained with DE-cad (red; to label cell outlines) and Vasa (green; to mark PGCs). (C) Quantification of the number of Vasa+ cells in control and pntaga embryos. (D) Quantification of the long, intermediate and short pnt mRNA levels in control and pntaga embryos using droplet-digital PCR. Measurements correspond to two biological and two technical replicates. In spite of no obvious differences in PGC numbers in both genotypes, pnt mRNA levels are decreased in pntaga embryos. Scale bars: 50 μm. Related to Fig 3. (TIF) [file pgen.1011809.s004.tif]

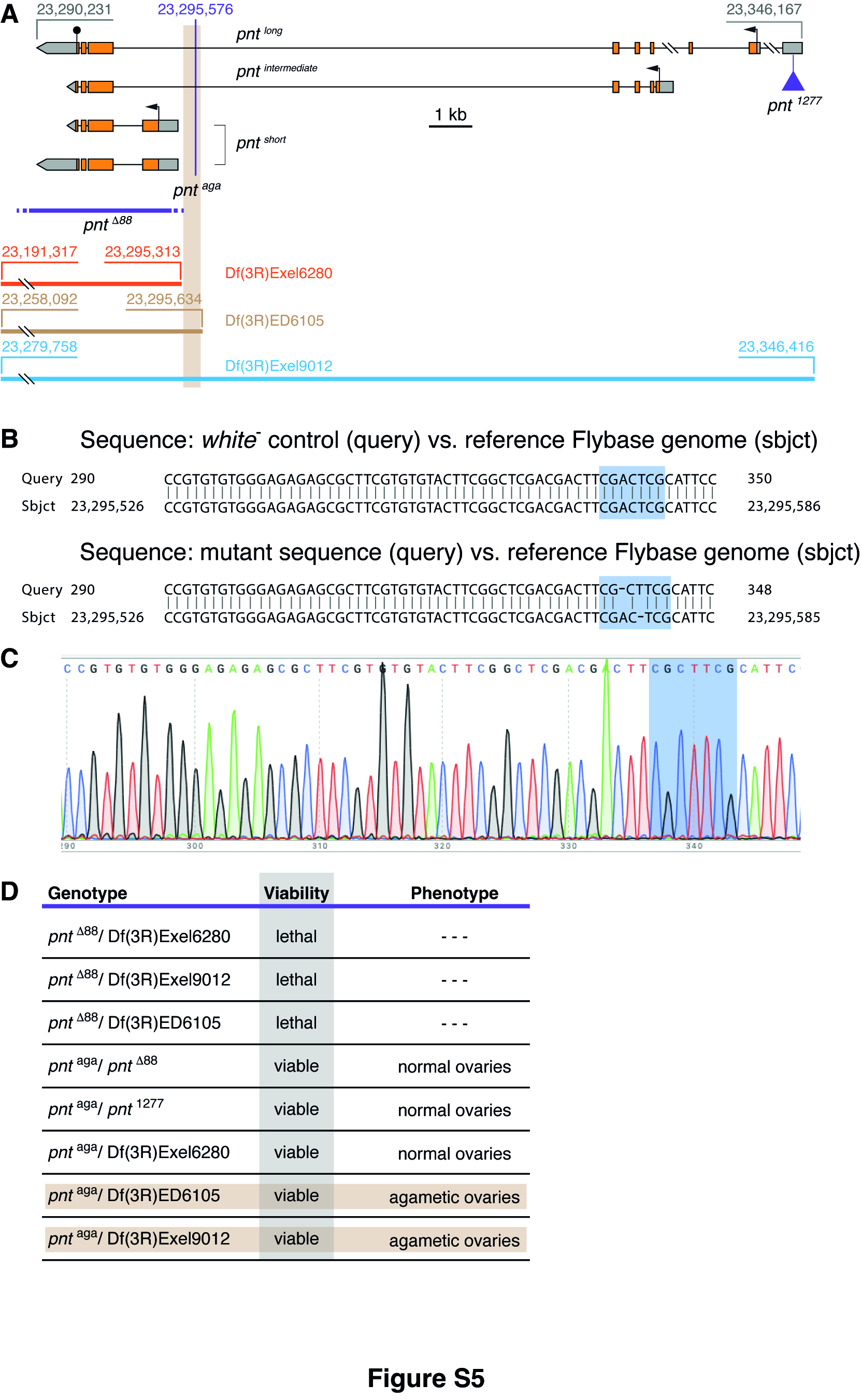

Supplement: S5 Fig — (A) Scheme showing the molecular characterization of pntaga, pnt·88, pnt1277 and the three deficiencies used to map pntaga. Numbers refer to genomic coordinates according to FlyBase (release FB2024_02). Mapping of pnt1277 and the pnt·88 deletion according to [11]. (B) Sequence of the region around the mapped pntaga mutation. The “subject” sequence corresponds to the reference sequence in FlyBase (release FB2024_02). The control “query” sequence is that of y w flies. The mutant “query” sequence is that of pntaga flies. (C) Chromatogram of the relevant region showing the two-base pair change in pntaga. (D) Table summarising the genetic characterization of different combinations of pointed mutants and deficiencies. (TIF) [file pgen.1011809.s005.tif]

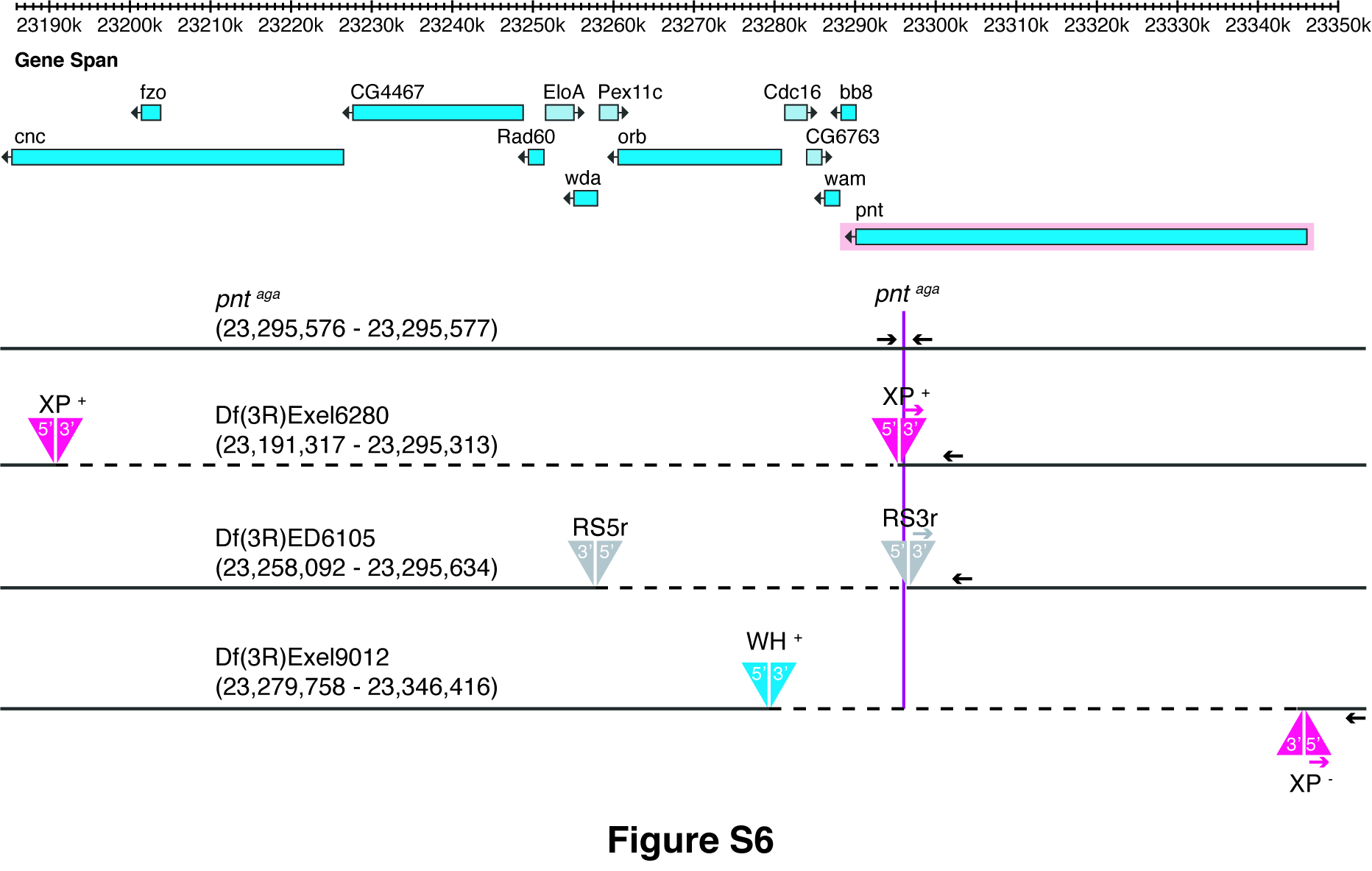

Supplement: S6 Fig — The precise mapping of Df(3R)Exel9012, Df(3R)Exel6280 and Df(3R)ED6105 was aided by the transposons used to generate them in the first place. Numbers in parenthesis correspond to the genomic coordinates of the breakpoints. Also shown are the genomic position of the primers used to map pntaga and the genomic coordinates of the two-base pair substitution found in this mutant. (TIF) [file pgen.1011809.s006.tif]
