# Supplementary material for: ETS transcription factor pointed controls germline survival in Drosophila
Source: PLoS Genet. 2025 Aug 25;21(8):e1011809. doi: 10.1371/journal.pgen.1011809 (PMC12407541; doi:10.1371/journal.pgen.1011809)
Supplement: Attachment — Submitted filename: Rosales_Nieves et al PLoS genetics rebuttal_letter.pdf [file pgen.1011809.s009.pdf]

Please convey our thanks to all the reviewers. Their comments have without doubt helped improve our MS and they indicate a fair amount of serious work from their part (and for free!). It is not that often that one gets these high-quality reviews.

Reviewer #1: The manuscript by Rosales-Nieves et al. highlights a novel role for the ETS transcription factor pointed (pnt) in gametogenesis and reproduction. In Drosophila, the EGFR signaling pathway is known to regulate primordial germ cell (PGC) numbers during female gonad formation, and pnt adds an additional regulatory layer to this process. The authors demonstrate that pnt is expressed in larval and adult gonads and is essential for gametogenesis.
They identified a novel mutant allele of pnt that results in germline loss and the development of agametic gonads. Interestingly, RNAi-mediated downregulation of pnt produces an opposing phenotype, with increased PGC numbers. Sequence analysis was conducted to identify genes affected by this mutation, and the results were compared with previously published data for other pnt mutant alleles. The authors found minimal overlap in differentially expressed genes among these mutants, suggesting that the various pnt isoforms have complex and specific roles in distinct cell types.
This manuscript is well-written, with excellent figures, robust data analysis, and clear conclusions that describe a previously unknown function of the pnt gene. With some revisions and additional experiments, I strongly recommend this paper for publication.

Major Comments:
1. To strengthen the claim that pnt acts in the soma to control germline survival, the use of an additional RNAi line is necessary. The choice to include a second TRIP line for downregulation experiments was commendable, but I recommend using Bloomington stock #35038 from the TRIP collection. This RNAi line, inserted into the VALIUM20 vector, is widely used due to its higher efficiency and lower off-target effects compared to older VDRC Vienna stocks. This line can effectively target both somatic and germline cells. Validation of RNAi efficacy via qPCR is also essential to confirm knockdown efficiency. Furthermore, this new RNAi line could be used for targeted downregulation of pnt specifically in germ cells, addressing whether the observed germline phenotype is consistently reproduced. Since Liu et al., 2010 also used a VALIUM20 RNAi line, testing with the TRIP RNAi line would help verify the phenotype and ensure robustness in the conclusions.
We have analysed the HMS01452 line (#35038 from the TRIP collection) expressed in the gonads under the c587-Gal4 driver. The c587>pnt HMS01452 combination gave the expected result, i.e. significant PGC overproliferation. This has been added to the text and to Fig. 2.

As for the validation of the RNAi reduction of *pnt* isoform levels. We have used ddPCR to quantify pntS, I and L in the three RNAi lines but without success. We cannot detect consistent reduction of mRNA levels in any of the conditions in spite of getting strong PGC phenotypes and after confirming that the 3 lines gave rough-eye phenotypes when expressed in the developing eyes using the GMR-Gal4 driver (see attached pictures for lines KK100473 and JF02227; both pictures have one control head and two RNAi samples). We believe that the larger number of *pnt*-expressing cells of the gonad (see Fig. S1) compared to the few cells expressing c587-Gal4 masked the RNAi effect on *pnt* transcription in the *pnt* RNAi gonads.

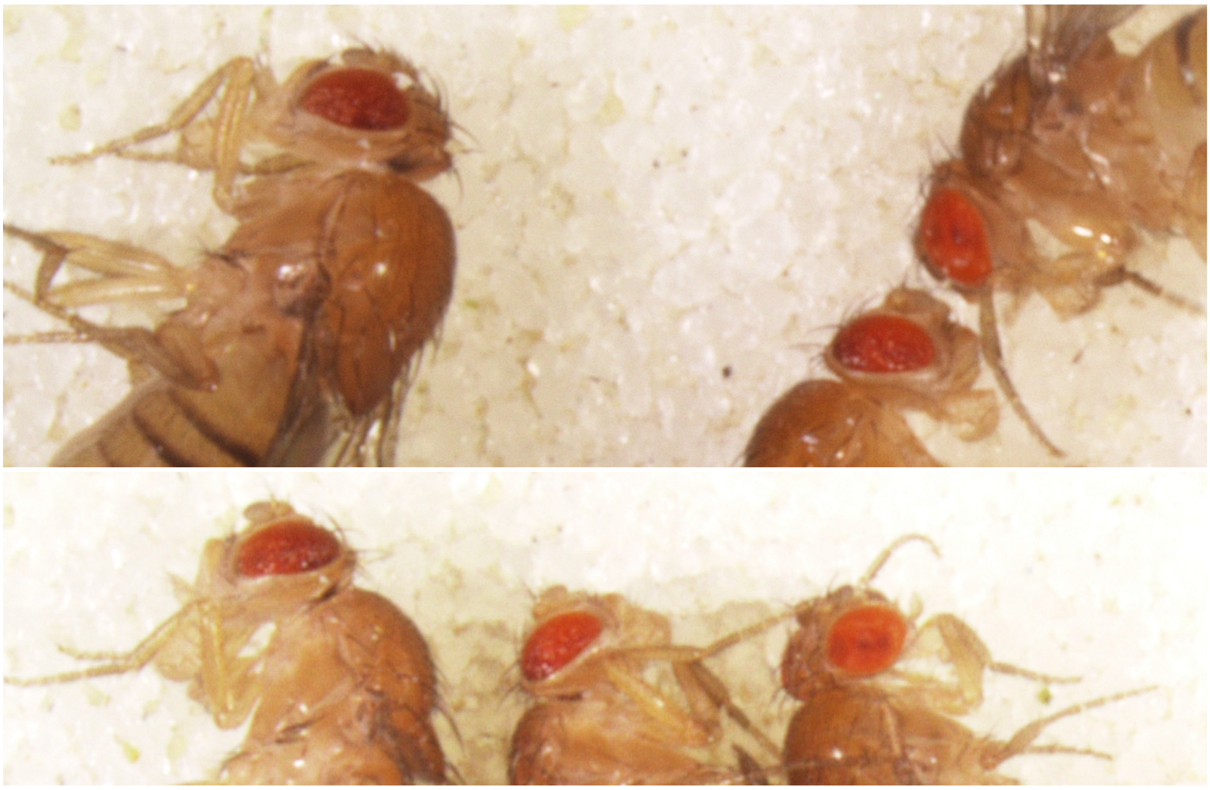

2. To support the hypothesis that pnt isoforms are regulated via an autoregulatory loop, I suggest measuring and comparing the expression levels of pnt isoforms not only in pntaga mutants but also in RNAi mutants that show distinct germline phenotypes. This analysis would provide direct evidence for an autoregulatory mechanism and link the observed germline phenotypes with changes in isoform expression levels.
This is an interesting suggestion, thanks. We have generated pntAGA/Df flies overexpressing either UASt-pnt^{short} (without a Gal4 driver) or c587-Gal4>UASp-pnt^{intermediate}. In both instances, we find an increase in pnt^{long} transcription, confirming our feed-back loop hypothesis. These results have been added to the text and to Fig. 4H.

3. The claim that cell death occurs exclusively in germ cells is not entirely convincing, as intermingled cells also appear to be affected. Can the authors confirm whether intermingled cells are undergoing cell death? This is critical, as intermingled cells are a key source of EGFR signaling, and their loss could disrupt germ cell differentiation and survival. Clarifying this point with appropriate markers or staining would greatly enhance the interpretation of the results.
The reviewer is right. We have looked at the expression of the DPC1 marker (it labels cells in the execution phase of apoptosis) in pntAGA gonads and, at the same time, labelling the intermingled cells. We did not see an increase in IC apoptosis in the mutant condition while we could see and obvious increase in PGC death. This has been added to the text and to Fig. S3.

4. The cause of germ cell loss in the ovary requires further exploration. Given the phenotypic parallels with EGFR signaling and the demonstrated interaction between pnt and this pathway, additional validation would strengthen the argument. Rescue experiments or analyzing reporter expression in the larval ovary could establish a direct link between pnt and EGFR signaling. Additionally, investigating other candidate signaling pathways regulated by pnt would enhance the overall understanding of its role in germ cell survival.
We have used the expression of the a-dpERK antibody as a read-out of receptor tyrosine kinase activity in controls, c587>pnt RNAi and pntAGA. We have found that both the RNAi condition and the pntAGA/Df combination decreased dpERK staining and hence RTK signalling. Considering that pnt is a known downstream

component of the EGFR pathway, we take this result as a demonstration that both experimental conditions are loss-of-function situations of EGFR signalling. This has been added to the text and to Fig. 4C

Regarding the study of additional signaling events putatively regulated by pnt, it would indeed be very interesting to look at other pathways, but we believe this is outside of the scope of our work.

Minor Comments:

5. Line 87: Clarify whether the sentence refers to the yan gene or the Yan protein.
Done.

6. Lines 100–102: The statement about terminal filament cells (TFCs) and cap cells (CpCs) in the anterior gonad is inaccurate. The anterior region contains other cell types that differentiate later, not just TFCs. Additionally, CpCs originate from intermingled cells, which are typically not categorized as anterior cells. Please revise accordingly.
We have modified the text. It now reads “Third larval instar gonads house PGCs and precursors of several somatic cell types including the terminal filament cells (TFCs) and cap cells (CpCs), the interstitial cells — called intermingled cells (ICs) — and the swarm cells (SwCs; Fig. 1C)”.

7. Figure 1C: While the schematic of the ovary is well-illustrated, there are inaccuracies to address:
i) The anterior region where terminal filament stem cells (TFS) reside is not an empty space; it includes somatic cells like anterior somatic cells and sheath cells. ii) The number of inner germarial (IG) cells depicted does not align with reality; significantly more IG cells are present. iii) Germ cells should not be labeled as primordial germ cells (PGCs) in this stage. Germ cells in contact with the niche are germline stem cells (GSCs), and those further away are differentiated germ cells. Revise this schematic and corresponding text (e.g., line 200) to use "loss of germ cells" as a more accurate term.
The drawing has been corrected.

8. Line 115: Update the number of ovarioles. Reports indicate there are typically 18–21 ovarioles in D. melanogaster, not 16–18.
Changed.

9. Line 152: Explain why UASp-Tau::GFP was chosen instead of the standard pUASp-GFP.
We have added the words “to distinguish clearly cell shapes” to the original sentence.

10. Line 155: Provide more specific details on pnt reporter's expression pattern. In larval ovaries, the pattern is restricted to a subset of TFCs near GSCs and CpCs. In adult ovaries, expression is limited to the anterior TFC and the last TFC in contact with CpCs. Specifying these differences is important, since TFCs exhibit distinct signaling pathway expression patterns.
We have changed the text following the reviewer’s suggestions.

11. Lines 174–180: These sentences are unclear and need rephrasing for better readability and understanding
Done.

12. Cell Proliferation: Confirm cell proliferation using specific markers, rather than relying solely on cell counts. This would add robustness to the data.
We have used anti-PH3 to score germline cells in mitosis. The graph has been added to Fig. 2D.

13. Figures 3H and 4A: While germ cell-positive markers are absent in the germarium, some cells with large nuclei appear in the escort cell region. Could the authors comment on the identity of these cells?

I am afraid we cannot. They most likely are Escort Cells, as they do not appear to have a follicle cell or follicle cell precursor shape. But we have not stained this agametic ovaries with escort cell markers.

14. Line 196: Clarify what n refers to in experiments—does it indicate the number of flies or ovaries analyzed? This specification is critical for understanding experimental design.
n here refers to number of gonads analysed. This has been clarified in the text.

Reviewer #2: The work of A. Rosales-Nieves et al. focuses on a genetic study of Pointed transcription factor role in the development of gonads in Drosophila. The research highlights two main observations. On the one hand, the expression levels of pointed are critical, as a significant loss leads to an increase in primordial germ cells, while a more limited loss results in their disappearance. On the other hand, the results suggest that pointed isoforms regulate each other. This work needs to be further consolidated before considering its publication. Moreover, its relevance for a large audience is not entirely obvious to me.

1. The increase in the number of PGCs is observed in two different RNAi constructs, but these transgenes target highly overlapping sequences, which does not rule out the possibility of a common off-target effect. Authors should find a way to exclude it. Moreover, a control RNAi (white, yellow, luc…) would be better rather than just comparison to Gal4 for the negative control.
Please see our response 1 to Reviewer 1 above. We have also done the controls with white RNAi, made with the same Valium20 plasmid and inserted in the same position as the new pnt RNAi line HMS01452.

2. Conversely, the aga mutant results in the absence of PGCs. However, according to Figure S5, it does not show a phenotype with the null mutant delta88, while it fails to complement larger deletions. The simplest interpretation would be that this allele (or another mutation on the same chromosome) affects another gene covered by the deficiency. Moreover, the authors provide no explanation regarding the origin of this mutation. If it originates from a genetic screen where flies were mutagenized, the existence of other mutations is likely. I am fully aware that the authors achieved rescue of this allele with transgenes, but the overall picture lacks coherence. Could it be that the phenotype is related to the concomitant loss of pointed and another gene?
We show that pntAGA does not give a phenotype over a null allele. This could be interpreted because there is interaction (transvection?) between both chromosomes. Hence, the wildtype pntS promoter from the pntdelta88 allele could activate pntS from the pntAGA chromosome, thus ensuring pntS levels. In addition, pntL would be transcribed from the pntAGA chromosome itself. Thus, the absence of phenotype. This trans-activation has been already observed for the pointed locus (Scholz H, Deatrick J, Klaes A, Klämbt C. Genetic dissection of pointed, a Drosophila gene encoding two ETS-related proteins. Genetics. 1993 Oct;135(2):455-68).

pntAGA was found by chance on a chromosome bearing a mutation on the tropomyosin II gene. We cleaned the chromosome (that is why we realized the phenotype was not due to tropoII but to another hit on the chromosome). And yes, the reviewer is right in the sense that the chromosome could we carrying second hits. But even in this scenario, proper pnt transcription is absolutely essential, as shown by the rescue experiments. Because of all of the above, we disagree that the “simplest interpretation would be that” a second hit combined with the pntAGA mutations is causing the phenotype. In our view, our original results plus the new data added to this second version of the MS strongly suggest that a novel combination of pnt isoforms is what generates the mutant phenotype.

Finally, we do not understand what the reviewer means here by "lack of coherence". Unless we were to re-create exactly the same damage to a chromosome of completely independent origin (and even then, we could not rule out the possibility of a second hit enhancing the phenotype), we believe we have done the appropriate controls to make sure that pntAGA creates a loss of function situation for the pnt locus, most likely not a null condition, because of the pntAGA + pnt RNAi experiment in which the gonads now show extra PGCs.

3. The authors' results suggest that the short isoform might activate the transcription of the long isoform. This hypothesis is easy to test: In their rescue experiment, does the expression of the short isoform increase that of the long one?
Again, thank you for the suggestion. Please see our response to Reviewer's 1 question 2.

4. The authors compare their differential expression results with those of other studies and deduce common targets. Does this correspond to a statistically significant enrichment? In other words, if random gene lists of the same size were intersected, what would be the expected number of common genes?
We have performed statistical analyses of the data in Fig. 5, which we think reinforce our conclusions, so thank you for the suggestion. This information has been added to the Results and M+M sections of the text.

Minor points:
- Scale bars are missing on many pictures.
We have checked all the figures and corrected the missing bars.

- Figure S1 shows that the locus seems to contain many enhancers leading to rather similar expression pattern but these results are poorly described. Is there an explanation for this? Could it be related to the fact that the isoforms seem to control each other?
We do not really have an explanation for this, more than saying that pnt is a large locus (50kb+) with seemingly many regulatory regions, with the added complexity of the feed-back loops we describe in the new MS.

5. Is there any evidence for a similar short isoform in mammals?
No, we have not found evidence of a pntS isoform in vertebrates. There is a homologue in sea urchin, though.

Reviewer #3: In this article, Alicia Rosales-Nieves and colleagues investigate the function of the ETS transcription factor Pointed during female gonad development in Drosophila.
Pointed belongs to the ETS domain family, a large group of transcription factors. Most ETS proteins are nuclear targets of Ras-MAP kinase signalling pathways, in particular upon activation of the EGF tyrosine kinase receptor.
The authors investigated the involvement of pointed (pnt) in the formation of the female gonads, following previous work indicating that silencing of somatic EGFR signaling causes overproliferation of primordial germ cells (PGCs).

They first used digital droplet PCR to precisely document the expression profile of the three pnt isoforms, identified as long, intermediate and short. In the L3 female larval gonad, only pnt short and long are expressed, while only pnt long is expressed in the ovarian tissues, including the germaria and early egg chambers. To further analyse pnt expression in female gonads, the authors used several enhancer-Gal4 lines and found that pnt expression is restricted to somatic cells of L3 female gonads and the ovarian GSC niche.
To assess the requirement for pnt during early oogenesis, they combine several RNAi lines whose expression is restricted to the somatic cells of the gonads and the germarium. In female larval gonads, somatic reduction of pnt expression leads to an increase in PGCS. In the adult, the authors report that somatic reduction of pnt expression in the ovary leads to larger germaria with more germ cells. They also show that reducing pnt in

somatic cells impairs their ability to correctly encapsulate the GC cyst.
The authors have isolated and characterised a new pnt mutation which, according to their results, specifically affects the development of PGCs from the L3 larval stage, with a significant decrease in the number of PGcs due to apoptosis in PGCs.
They further characterised the pnt aga mutation and found that it corresponds to a two-nucleotide change that affects pointed transcription.
Finally, they performed a comparative transcriptomic analysis between wild-type and pnt aa LL3 gonads and embryos.

Overall, this is a very comprehensive, multi-scale study with sophisticated and carefully chosen tools. The experiments are carefully made, and most of the data are presented in a clean nicely organized manner, well controlled and carefully quantified.

Main point:
This work provides new insights into the role of the EGF pathway and the ETS transcription factor, pointed in the control of PGC homeostasis in the female gonad.
However, the effect of the pnt aga mutation which is central in this manuscript is difficult for me to understand. On the one hand, molecular analysis seems to indicate a reduction in transcript levels for pnt long and pnt short.
On the other hand, the nature of the defects observed with pnt aga are opposite to those observed with pnt RNAi and are consistent with observations when the EGF pathway is inactivated: disappearance of CPGs with pnt aga when reduction of pnt by RNAi induces an excess of CPGs. Pnt aga seems to act as a gain of function. This is also consistent with the fact that pnt RNA i rescues the defects observed with pnt aga.
The reviewer's point is a fair one. We have changed slightly the text to make clear that our original observations and the new data in the second submission support the view that pntAGA gives rise to a new combination of pnt isoforms that induces PGC apoptosis. We prefer not to call pntAGA a gain-of-function mutation for several reasons. First, because dp-ERK levels are decreased in both, the RNAi and pntAGA conditions, likely indicating a loss of activity of the EGFR pathway; second, because pnt transcription is down-regulated in pntAGA; and third because the expression of a constitutively active form of EGFR in ICs reduces PGC numbers (Gilboa and Lehmann 2006; Matsouka et al. 2013), but does not cause an agametic phenotype as it happens in pntAGA.

Similarly, transcriptomic analysis of Pntaga embryos shows that 3 genes (hibris, cg1773 and 3 3-Hydroxymethyl-3-methylglutaryl-CoA lyase) are overexpressed, whereas these genes are underexpressed in pnt mutant embryos. This possibility is never mentioned by the authors.
We have identified 7 genes that are differentially expressed in pnt null embryos and in pntAGA gonads. All of them are down-regulated in mutant embryos compared to controls. Of these, only 4 show decreased expression while 3 are up-regulated in pntAGA gonads. Again, this piece of data indicates to us that the transcriptional regulation exerted by pnt is context-dependent rather than supporting a gain-of-function of pnt activity in the pntAGA condition.

The authors suggest that the difference in defects observed is related to the level of pnt transcripts. A large decrease, as with RNAi, would lead to an overproliferation of PGCs, whereas a decrease of 50% would have the opposite effect, the loss of PGCs. To test this possibility, the authors could reduce the efficiency of RNAi by lowering the induction temperature of gal4 or by using another gal4 line that is less expressed in somatic gonadal tissues. This can be monitored by ddPCR, as the authors have done for pnt aga. By focusing on gonad development at stage L3, the reduced efficiency in reducing the levels of short pnt and long pnt transcripts should lead to the loss of PGCs, as in the case of pnt aga.
As mentioned before, the comments by the reviewers and our new results have prompted us to change

slightly the text. We now emphasize the fact that pntAGA gives rise to a new combination of pnt isoforms. The consequences of this novel situation are dire for the germ line, which disappears. Thus, the main conclusion of the paper stands – that the ETS transcription factor pnt controls germ line survival in Drosophila.

We have also performed the experiment suggested by the reviewer. Utilizing the new RNAi line indicated by reviewer 1, we have grown larvae at 22, 25 and 29 degrees to test the effect of different levels of pnt knock-down on gonad development. As shown in the new Fig. 2, we obtained a gradation of the PGC proliferation phenotype. This result is one of the reasons why we believe pntAGA is not simple affecting levels of pnt isoforms but also generating a new combination of Pnt proteins that prevent germ line survival, a situation we may not be able to recapitulate by using simply an RNAi approach. We have looked at pntL and pntS expression in c587>pnt RNAi HMS01452 with ddPCR but, as explained in reviewer's 1 response 1, without success.

Minor points:
In the Figure 2, the increase of germline cell upon pnt rnai in somatic adult female gonads is difficult to distinguish, so a close up may help. We have added a new panel to the figure.

In the figure 3, with the panel I, the number of germ cells in embryos is very small this is probably due to the scale of the representation. It would be possible to modify the representation of these values with a different scale and insert them to complement figure sup S4.
Done.
